# Suspension of social welfare services and mental health outcomes for women during the COVID-19 pandemic in a peripheral neighborhood in São Paulo, Brazil

**DOI:** 10.3389/fpsyt.2022.897276

**Published:** 2022-09-15

**Authors:** Lenora Bruhn, Felipe Szabzon, Cristobal Abarca Brown, Daniela Ravelli Cabrini, Elisangela Miranda, Laura Helena Andrade

**Affiliations:** Nucleo de Epidemiologia Psiquiatrica, Instituto de Psiquiatria, Hospital das Clinicas da Universidade de São Paulo, São Paulo, Brazil

**Keywords:** global health, social support, gender equity/inequality, coronavirus, mental health, vulnerable populations, Brazil

## Abstract

During the COVID-19 pandemic, Brazilian urban peripheries have been severely affected both by the spread of the virus and by social, political, and economical dynamics, raising concerns about the psychological wellbeing and mental health of the population living in these areas. The pandemic broke out in a context of reduced public spending in social and health policies as well as in a process of erosion of social rights, fostering processes of exclusion and highlighting the association between austerity, the increase in poverty and inequality as well as in health and mental health problems indicators. This article presents the results of a qualitative participatory research that investigated subjective experiences in a peripheral neighborhood of São Paulo, Brazil, aiming to understand how contextual dynamics played a role in shaping mental health experiences during the COVID-19 pandemic. A multidisciplinary team of researchers worked closely with local volunteers trained to provide emotional support calls to neighbors of the community who signed up for the project. This article presents three ethnographic cases of women who had their routines strongly affected by the suspension of public and social protection services for the containment of the SARS-CoV-2 pandemic, leading to psychological suffering due to the increased demand of “domestic circuits of care”. We argue that within a context of austerity, the pandemic was remarkably harsh in urban peripheries and, specifically, for women with caring responsibilities. In addition to highlighting the pervasive “social protection gap”, the cases presented in this paper also reveals the unequal dynamics of the social reproduction work in several layers, which falls mainly on women's shoulders. The “crisis of care”, proposed by gender and feminist scholars, can contribute to understanding the psychological outcomes of the COVID-19 pandemic for these women.

## Introduction

The worsening of people's mental health is among the main concerns about the long lasting impacts of the COVID-19 pandemic (1–3). Given the emergence of a previously unknown virus and the guidelines to protect population from its patterns of fast spreading, people experienced great levels of insecurity, fear and many other types of distress (2, 4), frequently framed as mental health problems. Nonetheless, in various contexts around the world, the pandemic also overlapped economic and social crisis, accelerating processes of marginalization and social exclusion. This interaction of local and global dynamics is of foremost importance to understand how the spread of the coronavirus interacted with biological, cultural and social factors, and shaped the nature of people's distress and anguishes worldwide, often conforming synergistic environments in which health and social conditions mingled in a process that has been referred to as “syndemic” rather than strictly as a “pandemic” (5, 6). This contextual interaction questions the automatic association between the COVID-19 pandemic and its psychological outcomes. To think about this contextual interaction on mental ill health and wellbeing, Rose et al. (7) suggested moving toward an “neuroecosociality” framework that includes a biosocial approach of the immediate social environment of humans, who create and shape the “ecological niches” where they inhabit, being simultaneously shaped by them. The arrival of SARS-CoV-2 must, therefore, be situated in time and space and understood as an interaction of the spreading of the virus with economic and sociocultural dimensions.

Stuckler and Basu's (8) book, “The Body Economic”, brought together several examples on how fiscal austerity policies implemented in the context of the 2008 financial crisis have had effects on the worsening of the living and health conditions in several European countries. With a particular emphasis on mental health, Thomson et al. (9) have also explored the connections between austerity measures and the worsening of various epidemiological indicators. According to these authors, since the implementation of austerity policies in England that followed the financial crisis of 2008, gender and socioeconomic inequalities in poor mental health have increased, drawing attention to the fact that the first to bare the detrimental impacts of such policies are generally those who experience social disadvantages. In fact, very little research has been produced exploring the implications of feeble social policies to mental health in countries of the Global South to date (10, 11). Such countries are marked by greater inequality and face a pervasive gap in the scope of social protection nets, and have frequently been pointed to have an ubiquitous gap between mental health needs and the available service provision to address them (12–15). The fact that the pandemic has reached virtually all countries around the globe and, in one way or another, has had direct and indirect effects on the mental health of the global population, created new challenges especially for the Global South.

To explore the interconnections between the COVID-19 pandemic, its impacts on the mental health of the population, and sociocultural realities, Brazil may offer a relevant case. In fact, the country has made a significant increase in social investment during the 1990's and the first decade of the 2000's, which reflected on an expansion of access to many social policies, that sought to guarantee the rights provided by the 1988 Citizen Constitution (16, 17). Even though a comprehensive consolidation of a Welfare State was never achieved in the country, important attainments in several indicators on health, educational and socio-economic development were accomplished in this period (18). Also, a major mental health reform was also implemented in the country, ensuring greater access to services to poor populations as well as a shift from institutional to community care focusing in reducing the pervasive mental health gap (19, 20). Nevertheless, in recent years, these advances that had contributed to the reduction of many social inequalities have been systematically dismantled (10).

In Brazil, the COVID-19 pandemic broke out in a context of reduced public spending in social and health policies as well as in a process of erosion of social rights, which has been slowly consolidated in recent years, as a new logic of neoliberal government (21–23). Especially since the governments of Michel Temer (2016–2018) and Jair Bolsonaro (2019 – current), there has been a significant reduction in the scope of social policies that would be essential to contain the direct and indirect impacts of the health crisis that unfold from the pandemic. The greatest example of this process was the approval, in 2016, of Constitutional Amendment No. 95 (EC 95), which established a cap for public spending on social policies for the next 20 years, determining that budgetary changes in this sector would be restricted to an adjustment equivalent to the inflation rate. This measure has been pointed out as promoting a worsening in the living conditions of the Brazilian population and a setback in the fight to reduce inequalities, especially in a context of economic crisis such as the one experienced in recent years in the country (24).

Effectively, the pandemic accelerated the social consequences of dismantling social protection services, adding even more vulnerabilities and fostering processes of exclusion, highlighting the association between the reduction of public spending, the increase in poverty and inequalities as well as general health indicators (11, 25). Specifically, there was an increasing concern about the contingencies that this scenario would create to the mental health of the population (26). Several reports of the worsening of mental health conditions were published, with general guidelines for the functioning of mental health services, family caregivers, educators and primary care services (27–29). Thus, the main question that guided our research was which contingencies of the pandemic should be taken into consideration to better understand the psychological sufferings and distresses of marginalized populations living in contexts of poverty and exclusion. To understand local perceptions of emotional distress and suffering experiences, many authors have suggested paying attention to “narratives of distress” (30, 31). Narrative approaches can help to better understand how individuals talk and see their own mental health struggles and seek support (32, 33). Also, exploring first person narratives can help moving beyond a concept of mental health focused on disorders to one that comprehends mental health as a phenomenological experience, that takes place in time and space.

This article presents the results of a research project inspired in participatory action methods implemented through virtual meeting platforms and phone calls that investigated subjective experiences of dwellers of a peripheral region of the city of São Paulo, in Brazil, during the COVID-19 pandemic. Our objective was to explore how local residents experienced the circumstances imposed by the pandemic and to identify the main conditions underlying their anguishes and distresses. Some of the findings of this research have already been published elsewhere discussing the interfaces of mental health outcomes of the pandemic in contexts of urban insecurities (34). This article, more specifically, explores how reduced public spending, socioeconomic vulnerability and social reproduction responsibilities are contributing to widespread emotional distresses during the pandemic. As it will be seen in our results, the effects of the COVID-19 pandemic have had implications for the psychological wellbeing of those who experience social disadvantages and inequalities, specifically, of women living in the outskirts of the city of São Paulo. To contend with this assertion, stories of three women residing in the peripheral territory are presented, for whom the suspension of public social protection services during the COVID-19 pandemic resulted in situations of psychological suffering and feelings of emotional burden, often times so intense that disrupted their ability to cope with the stresses of life, realize their abilities, learn well and work well, and contribute to their community, all capacities defined under the concept of mental health.

## Methods

This research was inspired by Participatory Action Research methods (PAR). This approach sought to integrate the experiential dimension of first-person reports, expanding the validity and generalizability of our research findings (35–37).

The district of Sapopemba has a population of 284,524 people, most of them are self-declared black or brown, and a fifth of this population lives below the poverty line. This is equivalent to almost 60,000 people. Located in the south-eastern region of the city, Sapopemba is placed on an important route between central and eastern areas of the city. In addition to the precariousness faced by the local population, Sapopemba faced great difficulty in containing the spread of the COVID-19, being one of the regions of the city with the highest number of cases of contamination and death. The district is also characterized by a long history of social mobilization. This has ensured important achievements over the last 30 years in welfare services, such as day care for children, elementary schools, expansion of the public transport network and the implementation of a wide network of health services of the public health care system (SUS). All these achievements are related with struggles and social mobilization of local organized civil society movements (38).

In fact, most of the residents of the neighborhood live in small houses, often with several people sharing the same rooms of the house, and 21.58% of the houses do not even have running water and sewage. The percentage of informal workers in the region is also high, which led many people to have their income reduced with the closing of shops and slower circulation of people on the streets. These persistent vulnerabilities made the implementation of preventive measures harder and intensified the blockage of economic activities, helping to intensify the contagion and mortality caused by the pandemic in the district.

Members of the Center for Human Rights of Sapopemba (*CDHS*) and a team of researchers from the Section of Psychiatric Epidemiology of the Institute of Psychiatry, University of São Paulo Medical School (NEP/*IPq-HCFMUSP*) sought to create a community-based project aimed at strengthening ties of solidarity and emotional support among residents of the district of Sapopemba during the pandemic. Simultaneously, the initiative allowed to collect reports on the ways residents of the peripheral territory of the city of São Paulo frame, conceive and attribute meaning to their experiences of suffering in times of the COVID-19 pandemic.

Community leaders from the territory were appointed by the *CDHS* and other local social organizations to participate in the project as volunteers, making phone calls providing emotional support to other residents of the territory. The *NEP* team, together with the members of the *CDHS*, produced a semi-structured guidebook with general instructions to lead telephone calls that would be made by volunteers. The guidebook was structured as a support instrument to help the volunteers carry on the conversations. Volunteers received training in empathic listening and guidelines on how to conduct semi-structured interviews. To make the calls, each volunteer was sent a kit containing a guidebook, a notepad, a prepaid cell phone SIM card, a headset, and pens.

The project was publicized at the *CDHS* headquarters, where residents of the neighborhood interested in receiving a supportive call were able to provide their names and phone number. An enrolment list was circulated in social actions such as the distribution of food boxes to those in need. A list with 201 names and phone numbers was collected. We messaged all participants to verify their availability and make detailed information available as well as asking for those interested to sign a consent form. The information from interested parties was passed on to the volunteers, according to each one's availability. The volunteers were divided into two groups, which were followed up in weekly meetings by the research team through online platforms. Held between September and December 2020, these meetings were intended for reporting and discussing about cases heard by the volunteers, about the experiences of suffering that the people contacted by them were facing and how they could be supported. In addition, meetings were also spaces to create bonds between volunteers and researchers, to share questions and difficulties that could arise during the calls and for collectively discussing the experiences of suffering and caring arrangements. In this sense, volunteers played a dual role. On the one hand, they sought to strengthen ties of solidarity and provide emotional support to community counterparts. On the other hand, they reported the experiences heard from their perspectives and local repertoires on how suffering was being understood and experienced, as well as on possible referrals and ways to seek support to overcome the situations encountered. The project followed up a total of 18 people during this timeframe.

Meetings were recorded, transcribed, and analyzed using CAQDAS software (NVIVO 12). After transcription, the material was free-coded. Based on this coding, researchers discussed, in a multidisciplinary team (sociologists, social psychologists and psychiatrists), the main axes identified throughout the coding process, grouping the codes into themes that combine deductive and inductive approaches. Once the team has identified the relevance of the issue associated with the suspension of public services to women's mental health in the region, three ethnographic cases were selected as illustrative of this circumstance. The Research Ethics Committee of the Medical School, Clinics Hospital, University of São Paulo (CAAE: 46272221.2.0000.0068) approved the research protocol. All participants were informed about the purpose and scope of the study and the voluntary nature of their participation. Informed consent was obtained from all participants. As soon as community members signed up to participate in the project and received supportive calls, they received a message *via* WhatsApp to confirm their interest and a link to fill out an Informed Consent Form in digital format, as there was no in-person contact with any of the research participants and volunteers.

## Results

In addition to the lack of an energetic government response to the pandemic, existing social protection services suspended or reduced their activities, greatly impacting those who depend on them. When implementing the project and making available a list where people could enroll to receive emotional support, all entries were made by women. Perhaps one of the possible explanations to this result may be because registrations were carried out at the point where food boxes were delivered to families in need. Women contacted by the project in Sapopemba reported significant suffering resulting from the absence or reduction of assistance from the services and an increase in housework and caregiving activities. In this section we report the stories of three different women that illustrate how their mental lives were troubled by such circumstances.

### The case of I.: Suspension of in-person teaching in schools and day care centers

One of the women contacted by the project, I., lives in her own 3-room-house (bedroom, bathroom, and kitchen). According to her, she lives in the property with her three children, two girls from her first relationship, one aged 17 and one aged 10, and a baby from her current husband. The daughters are close to their father, although I. does not have any contact with her ex-partner. Her current husband, with whom she reports maintaining a very companionable relationship, has been in prison since 2019.

With the beginning of the pandemic, the necessary measures to fight COVID-19 resulted in the reduction of I.'s formal and informal support networks and the overload with housework and childcare was one of the reported sources of distress for her. Due to the suspension of activities in day care centers and school units, her three children started to stay at home full time. Before the pandemic, her mother used to help caring for the children. Nonetheless, this arrangement had to be suspended because she is elderly, and as I. had no place or anyone to leave the children, she had to leave her job. According to the report of the volunteer that contacted her,

She used to work in a bakery, but she had to leave so she could stay at home and care for the children. Previously, the youngest stayed at school, and after school she was taken care of by her grandmother, along with her little brother. Now, with the pandemic, I. must take care of her kids and had to stop working. She's having a hard time taking care of the kids on her own. [...] The 17-year-old girl has already finished school, now she is looking for a job. Looks like she's got it... Young Apprentice, you know? She's going to start the Young Apprentice service, which I believe it's paid, but I'm not sure. The 10-year-old girl is still studying. Now I. stays there with her 10-year-old girl and the little boy who is 1 year old. It's hard for her to go out to work, you know?

(W., project volunteer. Supervision meeting held on 10/22/2020).

Necessary social distancing measures during the COVID-19 pandemic have imposed heavy burdens on women in disadvantaged communities as household and care responsibilities have increased (39, 40). On the one hand, the suspension of in-person education in schools and day care centers put pressure on such private circuits of care. On the other hand, due to the risks of contagion and the greater fragility of the elderly population in relation to the coronavirus, I.'s mother, an important figure in the care of children before the pandemic, could not be present in her daily life as she did before. Historically, in these communities, informal support networks are extremely important to allow members of the household to pursue income revenues, and to “get by” other deprivations and precarious circumstances. Kinship and neighborhood social networks contribute to fostering the socioeconomic integration of individuals and to mitigate their conditions of vulnerability (41, 42).

Faced with the new scenario posed by the pandemic, I.'s family also had to adapt to remote education. Her 10-year-old daughter relied on her older sister to help with her with schoolwork. However, they had difficulties in doing online activities, because I. only had one cell phone and, sometimes, could not make it available for her daughter to study. During the period when the school remained closed, the girl could barely participate on online classes. Digital access during the pandemic made it possible for children and young people to continue their studies. However, a significant part of the youth, without adequate access to technology, either due to the lack of electronic devices (computers, tablets, and cell phones), or due to the lack of internet coverage in peripheral regions, had to interrupt their studies—or continue in an incipient way, as in the case of I's daughter. In the volunteer's words,

her sister helps her with the activities and she [I., the mother] also helps, but the 17-year-old sister is the one who helps the younger sister the most with school activities. She only has one cell phone in the household, which is hers, and she is not always there at the times her daughter needs it... she doesn't always have her cell phone available for her daughter to study, you know?

(W., project volunteer. Supervision meeting held on 10/22/2020).

With the onset of the pandemic, the suspension of welfare services overwhelmed families and communities. Under the lockdown, day care centers and schools were moved into people's homes, so the community had to absorb this demand, in addition to other responsibilities. This responsibility falls especially on women's shoulders, who frequently do most of this unpaid care work. The case of I.'s family also highlights the intergenerational cycle of care work among women in the domestic sphere, given that the three generations of women who ensured care work, particularly in the absence of the husband, or ex-husband, both of whom were absent in caring activities or in financially supporting their family.

Another of I.'s major concern was that, since the beginning of the pandemic, she had been unable to keep in touch with her husband because prisons were closed for family visits. They also couldn't communicate by letter, as the post office also stopped working. According to the volunteer who spoke with I.,

She is very sad because she has lost contact with him. Previously, they communicated by letter and visits. And all that got cut off, not even a letter anymore... an email system is being adapted, but she is not able to get in touch with him. She's feeling really sad, you know? [...]. She says she feels very sad and helpless. Very lonely [...]. She said she always goes to church to seek support. And that gives her strength in this situation because she is unemployed and has lost contact with her husband [...] [...]. She said they used to communicate more. When the pandemic started, you could really feel that she got really emotional, because she could neither visit nor write to him anymore. Then they completely lost contact. They're adapting a system *via* email; it looks like it's going to be digital now. But then they [jailers] read the conversations, or the message doesn't even get through...

(W., project volunteer. Supervision meeting held on 10/22/2020).

One of the main concern of families of imprisoned people was the news that the virus was spreading fast in prisons. Overcrowded conditions, poor hygiene, and lack of air circulation in prisons became even more worrying as the new coronavirus began to circulate within the walls of penitentiaries (43). During the pandemic, in addition to being vulnerable to the virus, without visits, inmates' families were concerned that their kins could be even more exposed to episodes of intramural violence and institutional abuse. Families of prisoners often have an important role in holding account of what happens inside detainment. The uncertainty of inmates' destinies generated numerous mobilizations of civil society. In this sense, I. claimed to be part of the association of family and friends of people in prison, through which she received information and had the support of counterparts in similar situation to hers.

I. reported not being afraid of contamination by COVID-19. Her biggest concerns were her financial situation, the overload with housework and childcare, and the lack of communication with her husband. According to the volunteer who kept in contact with I.,

The biggest problem is the absence of her husband and the children who are staying at home now, because she has changed her routine and is feeling overwhelmed. Those have been her concerns [...] and also because she feels she's alone in the battle, you know? (W., project volunteer. Supervision meeting held on 22/10/2020).

For the volunteer, I. pointed her children, mother, and religion as important supports. Although she remained unemployed during the period that we follow up supporting her with the phone calls, she had managed to re-establish the communication channel with her husband through letters, which brought her much relief. Also, her eldest daughter had started working, and I. received the Emergency Aid from the government.

The suspension of social welfare services, resulting from the social distancing measures, unbalanced the already weakened social protection networks to guarantee survival arrangements and to ensure that needs could be met. The absence of these services increased the demand on domestic circuits of care, especially overloading women of the poorest strata of the population, putting them in situations of intense stress and psychological suffering.

### The case of E.: Interruption of care structures for children with disabilities

In conversations with other women, similar difficulties arise. E., 40, is a housewife who is married and has two children, a teenage boy diagnosed with an autism spectrum disorder, and a 2-year-old girl. They live in their own brick-built house. At the beginning of the pandemic, her husband was fired, and their financial situation became one of the family's key concerns. He got a few side hustles at the same company he used to work, as well as independently producing and selling a car wheel cleaning product. E. is evangelical and relies primarily on her religion and church mates as a support network. According to the volunteer who kept in touch with her, when E. “*[...] needed to find strength and in moments when she was sad, she went to church and felt better, she got happy. For her, going to church was what got her healed*” (V., project volunteer. Supervision held on 10/10/2020). During the pandemic, while religious temples were closed to the public, E. said that she often prayed to talk to and ask God for help.

Before the pandemic, her son, who was in elementary school, was also accompanied by a social support center for people with disabilities, whose projects are carried out in partnership with the Municipal Secretariat for Social Assistance and Development. During the pandemic, however, the space remained closed for a few months. The loss of the support to the child was one of the reasons of major concern and stress for the family. The lack of this structure during school hours had a major impact on the dynamics of family care organization which also led to changes in behavior that the young man started to show. According to the volunteer who had contact with E.,

There are four people living in the house, herself, her husband and their two children. [...] when I asked her about how she would describe her life, how she was in this moment of pandemic, she just said: “very difficult”. Then she started to cry. And that's when she talked about her husband who lost his job, about her son who has this mental condition and the loss of the support from *Cantinho da Esperança* (“Little Corner of Hope”, a Social Support Centre) where he was cared for. There, he was followed up by a psychologist, interacted with other people, this was very good for him. Now his condition has deteriorated, and his behavior has changed. The support centre stopped functioning during the pandemic, E. said it might come back now, but she's not sure yet. She said that when she got married, she was 23 years old, in all that time, until today, she had never been through a situation like this. Now that her husband lost his job, they have almost nothing to eat at home. [...]

(V., project volunteer. Supervision held on 10/10/2020).

According to the volunteer who kept in touch, E. reported that both housework and childcare “was being a burden, [and that] she was feeling very bad and overwhelmed”. Because of this, “she and her husband fought a lot” and, occasionally, the husband took the boy with him “to sell the cleaning product, so he could also leave her a little more relaxed at home with the little one”.

We kept in touch with E. for 2 months, in late 2020. At the end of the year, when some services were gradually returning to function, E. reported feeling relieved by the return of her son to the activities promoted by the Social Support Center, even though the hours remained reduced. According to the volunteer,

*The Cantinho da Esperança* (“Little Corner of Hope”) will reopen from Monday to Thursday, from 1 p.m. to 3 p.m.; on November 4th. He will get a COVID test to return to the in-person service. There was a moment when E. said she was getting very unnerved in the pandemic, when her son got more aggressive. Then she took him to the doctor, and his medication was increased. Now he is taking four medications. Before he was only taking two. She said that he is getting better, but very often she repeated: “I can't trust my son anymore because he was very nervous and wanting to hit the 2-year-old little sister”. E. said that she keeps thinking: “they are there in the living room while I am here in the kitchen, the two children are alone”. She is feeling calmer now because of the return of the centre, even if it is for a short period of time. I see that she spoke more calmly now. In the beginning, on the first call, it was something that shook her a lot, the son's behavior. She says that with the pandemic, all other cases that were not related to COVID-19 were no longer a priority.

(V., project volunteer. Supervision meeting held on 10/29/2020).

The volunteer reported that E. felt overloaded with the increase in housework and childcare that resulted from the isolation guidelines due to the pandemic. Although religion was one of her main sources of emotional support, E. could not attend church because in these situations,

[...] who would look after the children, right? She couldn't take her son and because he was also very agitated, she stayed at home a lot more. So, I think this isolation at home, having all the children at home, the husband at home... all this created a certain feeling of despair in her. But faith was her refuge.

(V., project volunteer. Supervision meeting held on 10/10/2020).

Also, as it happened with I., E. had to stop working to be able to take care of the children at home. Both cases were of very poor families, for whom the loss of income could mean food insecurity and social vulnerability. According to the volunteer, “*she worked odd jobs when her son was at school, when he went to Cantinho da Esperança and when her daughter went to day care. So, as everything stopped, she ended up not doing it anymore. She stayed at home taking care of them*” (V., project volunteer. Supervision meeting held on 15/10/2020).

The daily problematic imposed by the domestic responsibility for care work during the COVID-19 pandemic in situations of absolute precariousness impacts on the lived experience of suffering for those who undertake this type of work. In E.'s case, in addition to the consequences that the closure of services had for her son's wellbeing, the lack of social protection resulted in great stress for herself, who lost her formal and informal support networks while the household and care burdens increased. Her suffering did not come from the fear of the virus or the quarantine itself, but from the domestic overload resulting from the lack of the social protection network she used to rely on, the family's food insecurity situation and from her son's suffering.

### The case of P.: Interruption of services in the centers for children and adolescents

P., another woman who was contacted by the project, lives in a rented house with her partner, a 10-year-old daughter and three teenage siblings. Even before the start of the pandemic, all family members were unemployed and relied on selling boiled sweetcorn and candies to passers-by on the street. With the beginning of the pandemic, because there was no one on the streets to buy the products they offered, they stopped selling them and began to depend on odd jobs at open street markets. Her middle brother was her main preoccupation on account of being a drug addict. According to P., he had just finished serving a reclusion sentence in a foundation for young perpetrators of infractions. He did not follow protocols to protect himself from the virus and regularly visited the drug den. Still, he had just found out that he'd gotten his girlfriend pregnant. Among the family members, only P. had received the Emergency Aid from the government, BRL 600.00 per month.^1^ The amount, however, barely covered the rent of the house where they lived, which costs BRL 500.00 a month and left them with a short margin for buying food or additional goods.

P.'s 10-year-old daughter was assisted by a Center for Children and Adolescents, an institution that aids children and young people at risk and social vulnerability. The center also had the activities suspended in compliance with preventive measures against the new coronavirus. The center used to ensure a meal for the children, but during the suspension of activities, it started to provide food boxes for the families of the children enrolled. By chance, the project volunteer who kept in touch with P. worked as a Pedagogical Coordinator in one of these Centers, located in another neighborhood. According to her report,

The procedure at the Centre where I work is like this: we have to fill out a form with the families' information, who will choose if they want to have in-person care for their children or if we keep working remotely. I If the child is going to return to in-person activities [...] we must schedule a day to do the serological test for COVID-19 on this child. If the result is negative, the child returns to in-person activities. But the mothers have already come here and said they don't want their children to come back because, if the child returns to in-person activities, the families will no longer get the food boxes. So many families are choosing not to come back because of the food. They understand that only one child is in the Centre and there are many more mouths at home to be fed. I have 55 families enrolled in my Centre—all opted to stay home.

(V., project volunteer. Supervision meeting held on 29/10/2020).

P. said that “*food boxes were a huge help*.” However, P.'s family was not given the option to decide between the return of in-person activities or to remain getting the food boxes. According to the volunteer, P. said that she had only been informed of the return and the consequent suspension of the food aid they had been receiving. In the volunteer's words, “*she was informed that her daughter would return to in-person care and that she would no longer receive food*.” Although she had no choice, P. said that, on the one hand, she considered it good for her daughter to go back to in-person care, because of the time she would have available to look for a job while her daughter was at the Center. On the other hand, however, due to the family's insecurity situation, P. was also feeling worried about their subsistence once they were relying on the meal kit delivered by the Center to ensure their basic food needs.

In this scenario, receiving the meal kit provided by the Center during the period the children were still at home impacts the household economy of the entire family. Failing to receive the kit means, as the volunteer explained, a loss in the household economy of the families, since the other members would no longer benefit. The child, in this sense, plays a dual role in the family, both as an offspring and as a mean of subsistence. The case of P. shows the position women occupy as a provider both in caretaking at the domestic sphere and to the family economics.

## Discussion

The social measures to contain the pandemic, such as isolation and social distancing, have led to the suspension of many social welfare services. As presented in the cases above, caring for children or dependent people relied pre-eminently on domestic labor, revealing and also deepening the structural care crisis that was already underway (40). Effectively, several authors have pointed out that those who took over the gap caused by the lack of social protection services during this time were mainly women. This was especially harsh in marginalized communities, namely for those living in urban peripheries and in contexts of poverty and exclusion (25, 44–47). The increase in the domestic burden harmed women not only in economic terms, but also had direct consequences for their structural position, accentuating pre-existing inequalities and also leading to daily uncertainties that affect their mental wellbeing (9), as shown in the cases of I., E. and P.

The discontinuity of public welfare services and the increase in caring responsibilities exacerbated a sense of helplessness experienced by the women contacted by the volunteers. The suffering, anguish and distress reported by them resulted precisely from the weakening of their subsistence arrangements and the consequent siege of their strategies to face precarious circumstances. Simultaneously, the lack of these fragile networks has jeopardized material and emotional dimensions of care. Despite its economic dimension, caring relationships also involve deeper personal and affective attributes.

The association between the mental health outcomes of the COVID-19 and the suffering experiences of women in the urban peripheries is ubiquitous in all cases presented. I., despite being married, performs as a single mother. During the pandemic, she was no longer able to rely on institutional support and family networks. Given that her mother belongs to a risk group, she could not be present in the daily care of her children as she did before. As she had no place or person to leave them with, I. had to leave her job, which further increased the family's material and financial vulnerability and made her feel unable to assure their subsistence and to address her children's needs. Likewise, E. also had to stop exercising paid activities because housework and childcare depended primarily on her own work. In her case, both school activities and the support center for people with mental disorders were suspended indefinitely. In addition, her youngest daughter's day care had also stopped activities. The family's financial situation and her children's wellbeing and health were at the center of her anguish, which caused her feelings of despair. P. was relieved when, months after the start of the pandemic, activities at the Center for Children and Adolescents in vulnerable situations where her daughter was enrolled resumed activities, albeit partially.

Indeed, gender and feminist studies have highlighted the central role posed by the moral dilemmas that arise from the responsibility of ensuring that care is adequately provided in the family milieu (48–50). The stories of the three women referred to the material and affective forces that derived from the responsibility of caregiving and the psychological suffering that unfolds from it. Flore et al. (51) considers that the social, intimate and affective experiences of caregiving, and the political contexts in which care labor is materially constituted, emerge as central elements to understand the many kinds of suffering lived by those who undertake these tasks. The emotional labor of care is also pointed by Fullagar and O'Brien (52) as central on women's sufferings. According to their research, sufferings were commonly articulated in relation to the responsibility of caregiving, mobilizing feelings of success/failure as mothers, workers and partners.

The cases here presented lead us to believe that the lack of formal welfare services intensified the sense of isolation, once care is increasingly relegated as an individual responsibility. Power and Hall (53) argue that governments are progressively advocating “personalization,” “choice and control,” and “autonomous and independent living” policies. In this scenario, care provision ends up depending on personal characteristics and individual capabilities. The neoliberal economy weakens and limits the strategies that people have to face their daily lives, falling on individuals' shoulders the responsibility of ensuring social reproduction and survival (41). Although social protection services were already insufficient and under dismantling, the COVID-19 pandemic has thrown the door wide open to the structural care crisis and the weaknesses of social protection systems, intensifying the appeal to individual solutions to deal with it (46). In the cases of the contacted women and their families, the accountability for care reproduces structural inequalities that are crossed by class and racial dynamics, which are remarkable in the Brazilian context (54). This pattern affects the most vulnerable women even in an intergenerational way. Cases like I.'s, for example, who has to leave her oldest daughter to take care of her youngest, generate chains of female care among underage women so that the adult woman can work in the formal labor market, while young girls miss the opportunity to study and play, both as important for personal and intellectual development.

In this scenario, marginalized communities and civil society organizations had to mobilized to face the “social protection gap”, and to ensure that the basic infra-structure to sustain life in times of the pandemic was offered (55). Churches, post offices and local associations were important resources to help people to keep going and to cope with the psychological struggles of daily life. Effectively, the social organization of care in Brazilian urban peripheries during the pandemic depended on networks structured around close social ties (relatives, co-religionaries, neighbors), participation in civil associations (social movement, Human Rights Centers or associations) and associative and religious practices. According to D'Andrea (56), this model was even more important during the pandemic. It was “without any decent state assistance” that “the peripheries fought the spread of the coronavirus, [using] a practice as old as it is fundamental for the survival of the poorest: solidarity” (p. 53). Historically, solving the issues of survival of underserved populations “almost always involves being on the streets mobilizing more extensive family, neighborhood and solidarity networks” (p. 48), which configures structures of opportunities and provides support for individuals to “get by” in order to mitigate material and affective vulnerabilities (41, 42).

Indeed, mutual support in contexts of church goers or the community of co-religionists, appeared as important supportive networks and were commonly mobilized in situations of suffering. Among the cases presented, participant E., an evangelical practitioner, sought emotional support and assistance in the local church in her community. In turn, I. resorted to the social movement of prisoners' friends and family. Through the movement, she was able to receive information about the functioning of prisons during the pandemic period, learn about and participate in political claims about the prisoners' rights, and also receive emotional support from other people who shared similar experiences.

Studies on health-related quality of life in Brazil during the COVID-19 pandemic detected higher prevalence of common mental disorders among females and low income groups, suggesting the improvement of mental health services (57–59). These findings corroborate our own results as they reinforce the association of the quality of life in contexts of vulnerability and poor mental health calling special attention to gender. Our ethnographic approach to understanding the meanings of women's psychological experiences can help in moving further with this suggestion, reinforcing that beyond formal mental health interventions, it is also important to ensure social rights and adequate policies supporting caregiving practices. In this sense, we assert that the State must implement policies in order to ensure that care can be adequately produced by the society. In so forth, practical competence and situated knowledge are fundamental for the development of appropriate public policies that address the needs to be met by care within territories. Thus, the elaboration of programs and public policies should be based on cultural heritage, proposals and understandings coming from social movements, commissions and councils that work at the ground level and are acquainted with specific issues and on locally feasible and suitable practices, and not in a way that overrides them.

The emerge of the pandemic demonstrated the extent of how we are “dependent upon and embedded within social relationships and institutions throughout the life course” (60), either to solve objective problems but also in subjective personal experiences in the everyday world. In addition to shedding light on our shared vulnerability in the social order, the pandemic has also exacerbated inequalities in access to social protection, demonstrating that the COVID-19 pandemic was, by no means, experienced in the same way across society. On the contrary, the way this period was experienced greatly depended on the individual's access to a range of social, relational, and material resources. In this sense, the strategies and means to face the harms, vulnerabilities and setbacks posed by the response to the virus dissemination should not be associated exclusively to personal characteristics, emotional tendencies or people's resilience capacities. Neither should these experiences be reduced to symptomatologic treatments. The cases here presented showed that the strategies to face many of the deprivations and precarious circumstances often rely on solid and strengthened (formal and informal) social support networks (61). In this sense, creating locally rooted institutional networks that allow thinking about the social organization of care for the sustainability of life can offer new possibilities for facing daily deprivations and precariousness, fighting inequalities and developing coping strategies for suffering and anguishes.

## Strengths and limitations

Participatory Action Research Methods (PAR) have been valued as a powerful tool to access the subjects' experiences and avoiding scientific paternalism (62, 63). By recognizing practices, discourses and positions as points of view about daily experiences, PAR methodologies allow the recognition of specific subjectivities in the localities where they are configured and developed facilitating a situated inquiry. The present study was based on an innovative research design that can benefit from these methodologies. Nonetheless, the pandemic and the adoption of security health protocols impeded a closer approach to important issues, such as the experiences of men on the peripheral areas of large cities and to explore in greater detail experiences related to race and ethnicity. These topics were only superficially touch by our inquiry and future studies would be able to explore that in greater depth.

Also due to social distancing protocols, we have relied in virtual technologies and online communication tools, either to exchange conversations with volunteers or for them to communicate with the people who received the phone calls. In addition to connectivity hurdles, there were also difficulties with hardware and adequate equipment to guarantee virtual communication. Also, the distancing protocols that imposed digital mediation, limited the possibilities of contact with people who do not have access to cell phones or internet connection, evidencing a pervasive dynamic of digital exclusion in contexts of poverty and deprivation.

## Conclusion

There is currently a broad international debate arguing that there is a pervasive gap in the provision of mental health services in different regions around the world, especially in low and middle-income countries, where there is an incipient investment in mental health policies. Several advocates of the so-called “Global Mental Health Movement” have pointed to the need for mental health services and a feeble availability of facilities (64). To repair this gap, it is often argued that countries need to expand the implementation of specialized services in the area (12, 65, 66).

The cases reported above point out, however, to an intertwined network of structural problems and experiences of suffering for which mental health services would have little to offer. Such circumstances can be rich reports to problematize the concept of gap itself, that has been mobilized over the last few decades. The cases allow us to reflect on two main questions. Firstly, we verify that what is often lacking to address the circumstances in which suffering is produced are not psychiatric and mental health services, but broader social protection policies that guarantee basic living conditions for marginalized groups, such as poor women living on the outskirts of São Paulo. The suspension of such services mainly affected already marginalized groups and characterizes in a particular way the sufferings experienced by these groups during the pandemic. This contingency challenges the universalistic association between COVID-19 and mental health outcomes. In so forth, it is necessary to consider the gap in a wider way, not only as a “mental health services gap”, but as a “social protection gap”. It is often due to the lack of social protection services, which ensure that basic social rights are met, that emotional distresses were created by the pandemic. In neoliberal contexts of the Global South, such as the Brazilian one, it is necessary to acknowledge barriers of access to social protection especially in terms of class, race and gender. The findings presented in this paper can bring some suggestions for Global Mental Health and its goals to target marginalized communities. The measures and guidance from international experts often represent a by-pass in this complex and rich network in which mental health is produced and cared for that goes far beyond specialized services. International guidance for mental health policy frequently relies on pre-formatted models for mental health care, often underestimating local support networks, that precede the development of more severe cases that will need formal mental health services. In this sense, nurturing models that increase social participation and social accountability can provide instrumental means to support policy formulation and to ensure that policies adhere to local forms of producing care and to address the concrete needs of the population.

## Data availability statement

The original contributions presented in the study are included in the article/supplementary material, further inquiries can be directed to the corresponding author.

## Ethics statement

The studies involving human participants were reviewed and approved by Research Ethics Committee of the Med School, Clinics Hospital, University of São Paulo (CAAE: 46272221.2.0000.0068). The patients/participants provided their written informed consent to participate in this study.

## Author contributions

LB, CA, and FS wrote the initial draft of the article. LB, CA, FS, DR, EM, and LA collected data and performed the analysis. LB, CA, FS, and DR conceived and designed the analysis. All authors were involved in reviewing the manuscripts, contributing to the interpretation of the results, and read and approved the submitted version.

## Funding

The research project Mental health in adversity: an ethnographic study of the experience of poor mental health in the favelas of São Paulo was funded by the State of São Paulo Research Foundation (FAPESP Grant number: 2019/17397-7). The Brazilian Council for Scientific and Technological Development supports LA (CNPq Grant 307933/2019-9).

## Conflict of interest

The authors declare that the research was conducted in the absence of any commercial or financial relationships that could be construed as a potential conflict of interest.

## Publisher's note

All claims expressed in this article are solely those of the authors and do not necessarily represent those of their affiliated organizations, or those of the publisher, the editors and the reviewers. Any product that may be evaluated in this article, or claim that may be made by its manufacturer, is not guaranteed or endorsed by the publisher.

## Footnotes

^1^Approximately 100.00 US dollars at the time.
